# Ischemic Area‐Targeting and Self‐Monitoring Nanoprobes Ameliorate Myocardial Ischemia/Reperfusion Injury by Scavenging ROS and Counteracting Cardiac Inflammation

**DOI:** 10.1002/advs.202414518

**Published:** 2025-01-22

**Authors:** Xiaobin Ma, Zhijin Fan, Jingyan Peng, Liming Nie

**Affiliations:** ^1^ Department of Cardiology Guangdong Cardiovascular Institute Guangdong Provincial People's Hospital Guangdong Academy of Medical Sciences Guangzhou 510080 China; ^2^ Medical Research Institute Guangdong Provincial People's Hospital (Guangdong Academy of Medical Sciences) Southern Medical University Guangzhou 510080 China; ^3^ Institute for Engineering Medicine Kunming Medical University Kunming 650500 China

**Keywords:** inflammation, myocardial ischemia/reperfusion injury, photoacoustic imaging, reactive oxygen species, ROS‐responsive nanoprobes

## Abstract

Precise and effective management of myocardial ischemia/reperfusion injury (MIRI) is still a formidable challenge in clinical practice. Additionally, real‐time monitoring of drug aggregation in the MIRI region remains an open question. Herein, a drug delivery system, hesperadin and ICG assembled in PLGA‐Se‐Se‐PEG‐IMTP (HI@PSeP‐IMTP), is designed to deliver hesperadin and ICG to the MIRI region for in vivo optical imaging tracking and to ameliorate MIRI. The peak aggregation of nanoprobes in the MIRI region is monitored by near‐infrared fluorescence and photoacoustic imaging. The maximal fluorescence and photoacoustic signals of the HI@PSeP‐IMTP group in the MIRI region rise ≈32% and 40% respectively compared with that of HI@PSeP group. Moreover, HI@PSeP‐IMTP effectively mitigates MIRI due to a synergistic integration of diselenide bonds and hesperadin, which can eliminate ROS and suppress cardiac inflammation. Specifically, the expression levels of p‐CaMKII, p‐IκBα, and p65 in the MIRI region in the HI@PSeP‐IMTP group demonstrate a reduction of 30%, 46%, and 42% respectively compared to that of the PBS group. Collectively, HI@PSeP‐IMTP provides new insights into the development of drugs integrating diagnosis and treatment for MIRI.

## Graphical abstract

Myocardial ischemia/reperfusion injury (MIRI) seriously threatens human life, and the corresponding monitoring and treatment require improvement. HI@PSeP‐IMTP, a highly biocompatible probe with advanced self‐monitoring capabilities, enables precise detection of ischemic area distribution and drug release via photoacoustic and fluorescence imaging. This study offers a comprehensive understanding of the probe behavior and efficacy, and shows great potential in alleviating MIRI.

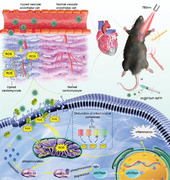

## 1 Introduction

Acute myocardial infarction is one of the major fatal diseases, with a high morbidity rate worldwide.^[^ ^1^ ^]^ Timely restoration of coronary blood flow plays a critical role in reducing mortality. However, this vital intervention can trigger myocardial ischemia/reperfusion injury (MIRI), characterized by cardiac inflammation and excessive reactive oxygen species (ROS), and further lead to heart failure.^[^ ^1^, ^2^ ^]^ To date, MIRI is still a formidable challenge in clinical practice. Despite significant progress in experimental research, where numerous treatments have demonstrated robust protection against MIRI, the translation of these promising findings into clinical practice has been frustratingly slow. This discrepancy may stem from the intricate and multifaceted nature of cardiomyocyte death mechanisms triggered by MIRI, as well as the limitations posed by single‐target therapeutic approaches.^[^ ^3^ ^]^

The multifunctional serine/threonine protein kinase, Ca^2+^/calmodulin‐dependent kinase II (CaMKII), with CaMKII‐δ being dominant in the heart, plays a pivotal role in cardiac function.^[^ ^4^ ^]^ When cardiac cells suffer from ischemic reperfusion injury, the phosphorylation levels of CaMKII‐δ undergo a marked surge, exacerbating MIRI and eliciting pathological inflammatory responses along with cardiac dysfunction.^[^ ^1^, ^4^, ^5^ ^]^ To address this issue, hesperadin emerges as a specific inhibitor of CaMKII‐δ, effectively quelling its overactivation and subsequent inflammatory cascade, thereby alleviating mitochondrial damage and diminishing ROS production in cardiomyocytes.^[^ ^1^, ^6^ ^]^ However, a small molecular compound is always characterized with short half‐life, rapid clearance in vivo, and poor target retention,^[^ ^7^ ^]^ including hesperadin. Consequently, subcutaneous or intravenous administration of hesperadin may cause its extensive distribution in vivo and non‐specific elimination by metabolic organs, markedly diminishing its concentration at the area of ischemic myocardiocytes and exerting adverse effects on normal organs. Furthermore, given the poor efficacy of single‐target therapies, multiple‐target strategies hold promise for more effectively restoring myocardial function after ischemic reperfusion injury.^[^ ^3a^ ^]^

Currently, all kinds of nanomaterials are being utilized as drug carriers to improve the diagnostic accuracy and therapeutic efficacy for MIRI, exhibiting remarkable efficiency in these regards.^[^ ^2^, ^8^ ^]^ Nevertheless, despite their promising potential, the clinical translation of these nanomaterials remains elusive primarily due to concerns over their unverified biosafety. Moreover, to optimize their performance, nanoprobes designed for both diagnostic and therapeutic purposes must undergo improvements in terms of precise targeting toward MIRI region, ROS response, self‐monitoring, and multiple‐target therapy.

In view of the above‐mentioned facts, it is imperative to devise a delivery strategy that integrates biosafety, precisely targeted delivery, controlled release, self‐monitoring, and multiple‐target therapy. Poly (lactic‐co‐glycolic acid) (PLGA), characterized by its low toxicity and biodegradability, has been a staple in encapsulating therapeutic agents for targeted therapy over the years.^[^ ^9^ ^]^ However, due to PLGA's lipophilic nature, it tends to be recognized and cleared by the mononuclear phagocyte system in the bloodstream. To deal with this issue, poly (ethylene glycol) (PEG) is usually employed as a coating to enhance the hydrophilicity of PLGA, thereby reducing clearance and prolonging circulation time.^[^ ^10^ ^]^ In clinical practice, the selective homing of drugs to the ischemic region is an open question. CSTSMLKAC, an ischemic myocardium‐targeted peptide (IMTP), emerges as a promising solution due to its excellent targeting ability toward ischemic myocardium, as evidenced by phage display experiments.^[^ ^11^ ^]^ Mechanistically, that cardiac troponin I (cTnI) is exposed to the extracellular environment of injured myocardium can facilitate IMTP's interaction with cTnI.^[^ ^11b^ ^]^ Therefore, decorating the surface of the delivery system with IMTP can significantly enhance its affinity for injured myocardial cells. Furthermore, selenium, an essential micronutrient in animals, plays a crucial role in various physiological processes.^[^ ^12^ ^]^ Given the abundance of ROS generated in injured cardiomyocytes, incorporating ROS‐responsive diselenide bonds into the delivery system can achieve a controlled release of therapeutic cargoes.^[^ ^13^ ^]^ Additionally, diselenide bonds can be oxidized to seleninic acid by ROS or reduced to selenolin, thereby contributing to ROS scavenging,^[^ ^14^ ^]^ which synergizes with hesperadin to mitigate inflammatory injury in cardiomyocytes.

Besides, it is significant to monitor the process of the drug‐delivery system homing in on the MIRI region, which is conducive to assessing the targeted capability of drug‐delivery system and the extent of MIRI. Indocyanine green (ICG), a powerful cyanine near‐infrared (NIR) dye, possesses exceptional tissue penetration capabilities, making it ideal for NIR and photoacoustic imaging applications.^[^ ^15^ ^]^ By integrating ICG into PLGA, we can dynamically visualize the distribution of the drug‐delivery system and gain insights into the scope of MIRI in real‐time. Notably, the combination of ICG and PLGA fosters intense *π–π* stacking interactions, setting off aggregation‐caused‐quenching (ACQ) effect.^[^ ^16^ ^]^ While the drug‐delivery system arrives in the area of MIRI, the microenvironment with high ROS triggers the degradation of the nanoprobes, subsequently prompting the incremental release of ICG. This response to the microenvironment can be self‐monitored by NIR and photoacoustic imaging.

**Scheme 1:**
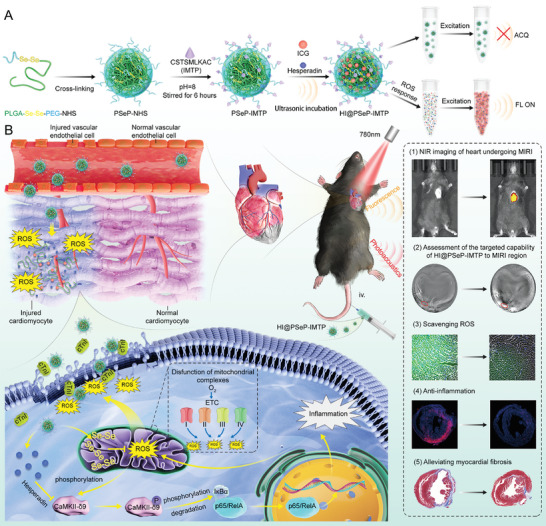
A) Schematic diagram of the preparation of a drug delivery system (named as HI@PSeP‐IMTP). B) Schematic diagram for HI@PSeP‐IMTP targeting MIRI region and tracked by NIR imaging system and photoacoustic imaging system in MIRI region as well as the underlying therapeutic mechanism of HI@PSeP‐IMTP.

Collectively, we design a drug delivery system, hesperadin and ICG assembled in PLGA‐Se‐Se‐PEG‐IMTP (HI@PSeP‐IMTP), which could target the region of MIRI for ROS‐responsive release of cargoes and real‐time self‐monitoring (**Scheme** **1**). Hesperadin and diselenide bonds coordinate the scavenging of ROS and the alleviation of myocardial inflammation, thereby facilitating cardiac repair.

## 2 Results and Discussion

### 2.1 Preparation and Characterization of HI@PSeP‐IMTP

**Figure 1:**
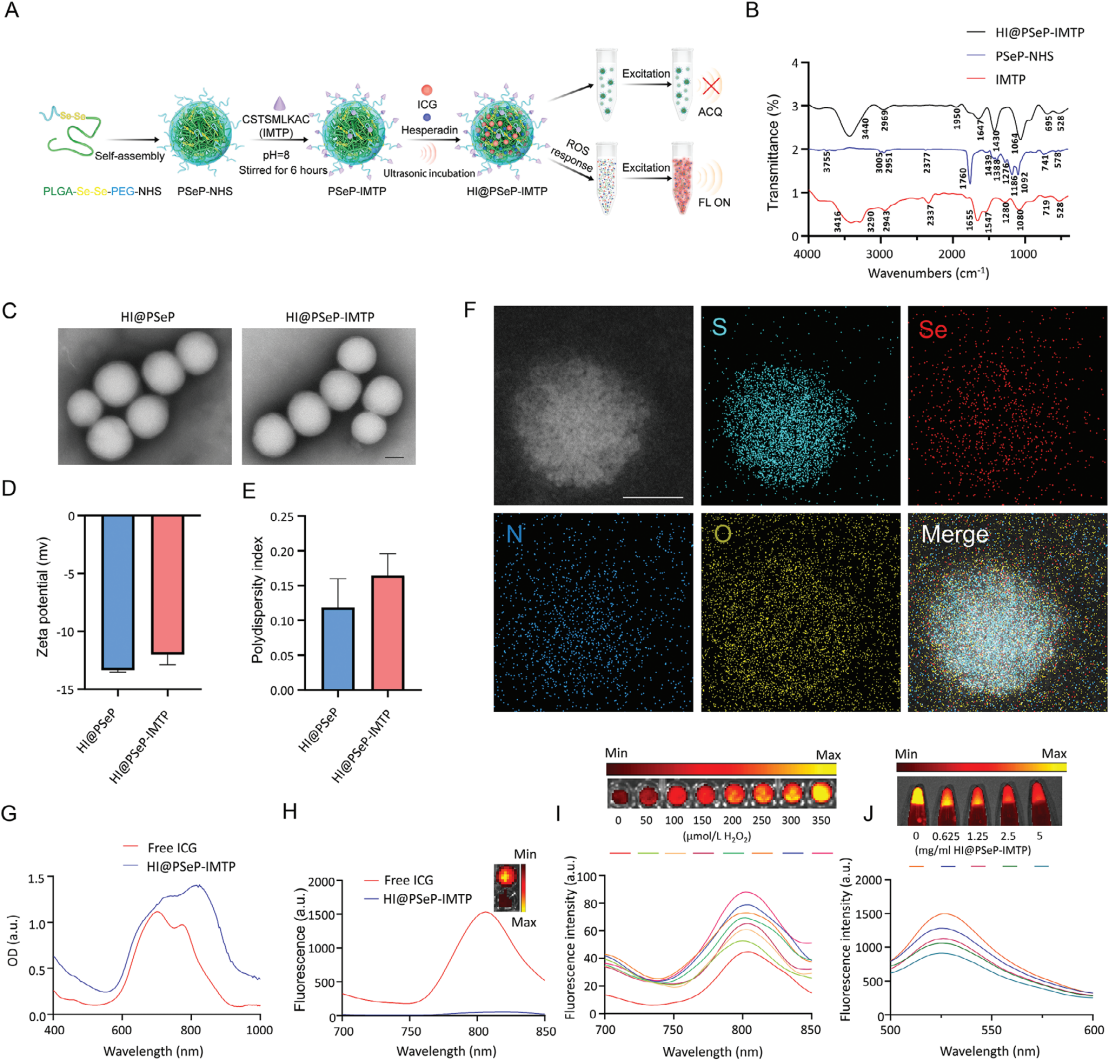
Preparation and characterization of HI@PSeP‐IMTP. A) Schematic illustration of the synthesis and property of HI@PSeP‐IMTP. B) FTIR was utilized to analyze IMTP‐labeled HI@PSeP‐IMTP. C) The morphology of HI@PSeP and HI@PSeP‐IMTP was monitored using TEM. Scale bar, 50 nm. D,E) The Zeta potential and PDI of nanoparticles were measured by Litesizer 500. F) Energy dispersive spectrometer mapping of HI@PSeP‐IMTP. Scale bar, 50 nm. UV–vis–NIR absorption spectrum G) and fluorescence spectrum H) of free ICG and HI@PSeP‐IMTP. I) Assessment of ROS‐responsive property of HI@PSeP‐IMTP. J) Evaluation of ROS scavenging capacity of HI@PSeP‐IMTP.

We successfully synthesized PLGA‐Se‐Se‐PEG‐NHS (PSeP‐NHS) nanoparticles by an emulsion solvent evaporation method. Next, we further adorned PSeP‐NHS nanoparticles with IMTP. Subsequently, hesperadin and ICG could self‐assemble into nanoparticles, which guarantees the ultimate synthesis of HI@PSeP‐IMTP (**Figure** **1**A).

To verify the successful integration of IMTP onto the surface of the nanoparticle, Fourier Transform Infrared Spectroscopy (FTIR) was employed to assess the spectral characteristics before and after the modification. The results showed the characteristic peaks of IMTP and PSeP‐NHS in HI@PSeP‐IMTP, which verified the conjugation of IMTP on the nanoparticles (Figure 1B). Meanwhile, a dynamic light scattering (DLS) experiment was applied, revealing that the zeta potential shifted from −13.37 mV (HI@PSeP) to −12.02 mV (HI@PSeP‐IMTP) after the modification of IMTP while these nanoparticles were monodispersed in PBS, with polydispersity index (PDI) values of ≈0.15 (Figure 1D,E). This finding underscores the successful attachment of IMTP to the nanoparticles. Additionally, we demonstrated that ≈65% IMTP successfully bound to the nanoparticles by Nanodrop 2000. The encapsulation amounts of hesperadin and ICG were detected by high‐performance liquid chromatography (35.56 µg mg^−1^) and microplate reader (50 µg mg^−1^), respectively (Figure S1A,B, Supporting Information).

Upon examination with transmission electron microscopy (TEM), the morphology and particle sizes of both HI@PSeP and HI@PSeP‐IMTP were determined to be close to a circle in shape and ≈100 nm in diameter, indicating their consistent nanoscale features. (Figure 1C). The results indicated that the modification of IMTP on nanoparticles exerted little effect on the nanoparticle size. To ascertain the definite composition of the key elements, energy‐dispersive spectroscopy (EDS) mapping was utilized and the results showed the coexistence of hesperadin (containing sulfur), ICG (containing sulfur), and selenium, which validated the successful encapsulation of cargoes in HI@PSeP‐IMTP (Figure 1F). Subsequently, we demonstrated that HI@PSeP‐IMTP was stable in PBS at 4 °C by DLS experiment, and H_2_O_2_ can promote the rapid release of cargoes from HI@PSeP‐IMTP (Figure S1C,D, Supporting Information).

Utilizing a microplate reader, we analyzed the absorbance and fluorescence intensity of both free ICG and HI@PSeP‐IMTP to discern the ACQ effect present in the latter. The results indicated that the fluorescence intensity of HI@PSeP‐IMTP was significantly lower than that of free ICG while the absorbance value of HI@PSeP‐IMTP was higher than that of free ICG (Figure 1G,H). Subsequently, we dissolved HI@PSeP‐IMTP in PBS possessing various concentrations of H_2_O_2_. As the concentration of H_2_O_2_ in PBS escalated, the release of ICG from the nanoparticles intensified, leading to a gradual enhancement of the corresponding fluorescence intensity (Figure 1I). These findings implied that HI@PSeP‐IMTP exhibited an ACQ effect, which could significantly impact the assessment of the targeting efficacy of nanoparticles and drug release in vivo. Beyond merely responding to ROS, HI@PSeP‐IMTP also possessed the capability to scavenge ROS. We used a ROS Assay Kit to detect the H_2_O_2_ clearance capacity of HI@PSeP‐IMTP. Our results showed that as the concentration of HI@PSeP‐IMTP rose, the corresponding fluorescence intensity declined, implying a heightened capacity for H_2_O_2_ elimination. (Figure 1J).

In summary, the drug delivery system, HI@PSeP‐IMTP, demonstrated excellent characterization, robust ROS‐responsive property, and effective H_2_O_2_ scavenging capability. These findings underscored its potential for in vivo cargo delivery, controlled release in the region of MIRI, and achieving combination therapy.

### 2.2 Targeted and Therapeutic Capabilities of HI@PSeP‐IMTP In Vitro

**Figure 2:**
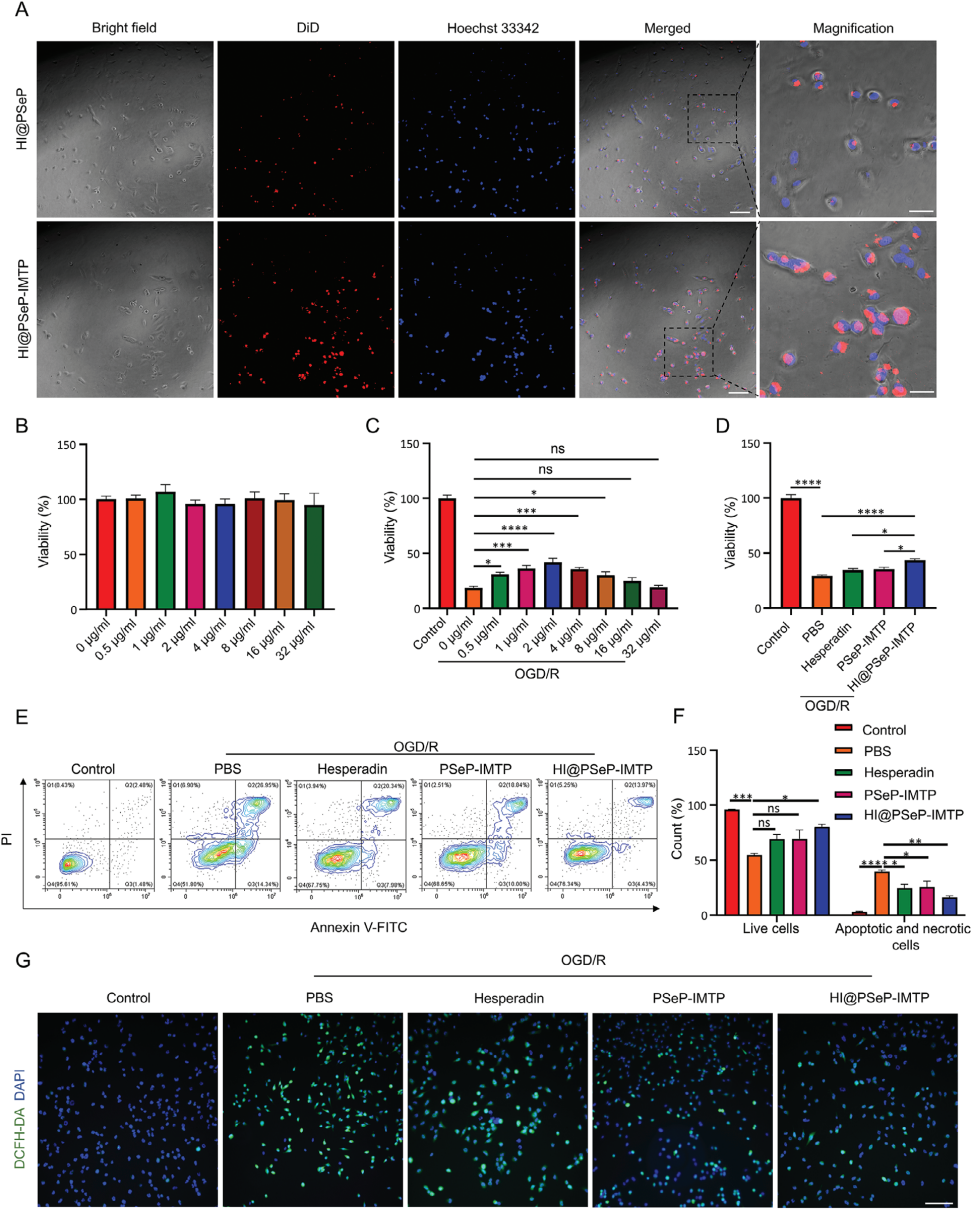
The targeted and therapeutic capability of HI@PSeP‐IMTP in vitro. A) Fluorescence imaging was employed to detect the targeted capability of HI@PSeP‐IMTP to AC16 cells subjected to OGD/R. Scale bar, 150 µm. Enlarged scale bar, 50 µm. B) Assessment of the cytotoxicity of HI@PSeP‐IMTP in different concentrations. C) Detection of the optimal concentration of HI@PSeP‐IMTP in improving the viability of AC16 cells undergoing OGD/R. D) Evaluation of the synergistic treatment efficacy of diselenide bonds and hesperadin. E,F) Effects of different treatments on apoptosis of AC16 cells subjected to OGD/R. G. ROS scavenging capacity of different treatments. Scale bar, 200 µm. The results were presented as the mean ± SEM (*n* = 8 for the monitoring of cellular viability; *n* = 3 for the apoptosis assay; ns, no significance, ^*^ *p* < 0.05, ^**^ *p* < 0.01, ^***^ *p* < 0.001, ^****^ *p* < 0.0001).

To investigate the targeted capabilities of HI@PSeP‐IMTP in vitro, oxygen‐glucose deprivation/reoxygenation (OGD/R)‐treated AC16 cardiomyocytes were incubated with 1,1′‐Dioctadecyl‐3,3,3′,3′‐Tetramethylindodicarbocyanine perchlorate (DiD)‐labeled HI@PSeP and HI@PSeP‐IMTP in a 96‐well plate. As expected, HI@PSeP‐IMTP exhibited a greater affinity for targeting OGD/R‐treated AC16 cardiomyocytes, showing more intensive DiD fluorescence in these cells, as detected by immunofluorescence technology (**Figure** **2**A).

Subsequently, cell counting kit‐8 (CCK8) assay was applied to assess the viability of normal AC16 cells 24 h after being incubated with HI@PSeP‐IMTP at different concentrations. The results showed that there was no significant cellular toxicity of HI@PSeP‐IMTP (Figure 2B). To further determine the optimal therapeutic concentration of HI@PSeP‐IMTP, CCK8 assay was used to discern the viability of OGD/R‐treated AC16 cells. Interestingly, the viability of these cells rose at the beginning and then declined with the increase of concentration of HI@PSeP‐IMTP. Importantly, 2 µg mL^−1^ HI@PSeP‐IMTP was verified as an optimal therapeutic concentration because of its better therapeutic efficacy (Figure 2C). Additionally, we further evaluated whether 2 µg mL^−1^ HI@PSeP‐IMTP possessed more effective treatment than its corresponding composition, hesperadin, and PSeP‐IMTP. Analysis by CCK8 assay and flow cytometry‐based Annexin V/PI cell apoptosis detection demonstrated that HI@PSeP‐IMTP could effectively decrease cell apoptosis rate and preserve the viability of AC16 cardiomyocytes, while hesperadin and PSeP‐IMTP showed a negligible effect (Figure 2D–F). Similar results were obtained through CAM/PI double staining (Figure S2, Supporting Information). Ultimately, ROS detection assay was performed, showing that OGD/R‐treated AC16 cells incubated with HI@PSeP‐IMTP produced less ROS (Figure 2G).

In conclusion, HI@PSeP‐IMTP possessed excellent binding capability to AC16 cardiomyocytes suffering from OGD/R and exerted a positive effect on protecting these cardiac cells.

### 2.3 Binding Capability of HI@PSeP‐IMTP In Vivo

The delivery of cardioprotective drugs to the region of MIRI primarily relies on the enhanced permeability and retention (EPR) effect.^[^ ^17^ ^]^ However, the EPR effect only exists for a short time after myocardial injury, which cannot supply sufficient protection to injured myocardial cells.^[^ ^8^, ^13^, ^17^, ^18^ ^]^ As a biomarker of myocardial injury, cTnI is exposed to the extracellular environment while myocardium suffers from ischemic attack, which makes injured cardiac cells susceptible to be hitched by IMTP. This preferential affinity for ischemic myocardiocytes could cause incremental retention of HI@PSeP‐IMTP in the region of MIRI.

**Figure 3:**
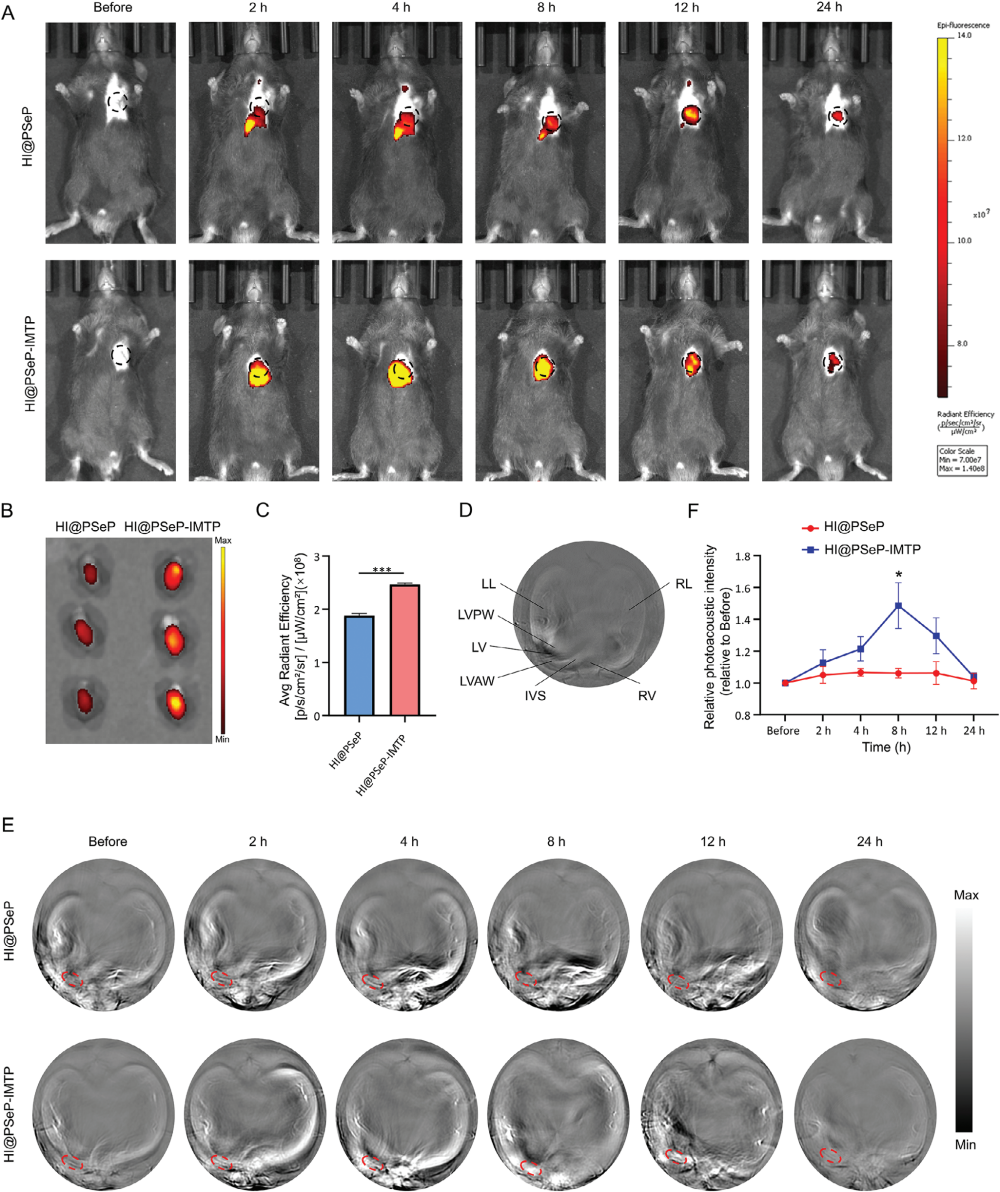
Binding capability of HI@PSeP‐IMTP in vivo. A) NIR fluorescence imaging was applied to discern the aggregation of HI@PSeP‐IMTP in the heart suffering from MIRI in vivo. B,C) Assessment of the targeted capability of HI@PSeP‐IMTP to MIRI region ex vivo. D) Cross‐sectional image of the heart in a normal mouse was observed by PACT. LL, Left Lung; RL, Right Lung; RV, Right Ventricle; IVS, Interventricular Septum; LV, Left Ventricle; LVAW, Left Ventricle Anterior Wall; LVPW, Left Ventricle Posterior Wall. E,F) PACT was employed to detect the binding capability of HI@PSeP‐IMTP to the MIRI region. The results were presented as the mean ± SEM (*n* = 3; ^*^ *p* < 0.05, ^***^ *p* < 0.001).

To elucidate the binding capability of HI@PSeP‐IMTP, mice suffering from MIRI were administered with nanoprobes through the tail vein 24 h after modeling. NIR fluorescence imaging indicated that HI@PSeP‐IMTP was more feasible to be hitched to the area of MIRI (**Figure** **3**A). Importantly, the signal‐to‐background ratio (SBR) of precordium reached a peak on the eighth hour and then gradually decreased in the HI@PSeP‐IMTP group, whose SBR valued ≈1.43 times that of HI@PSeP group at this time. To further ensure the targeted capability of HI@PSeP‐IMTP, we extracted major organs of mice on the eighth hour and NIR fluorescence imaging technology was conducted. The results demonstrated that the fluorescence intensity of ischemic myocardium in HI@PSeP‐IMTP group rose by ≈32% compared to HI@PSeP group (Figure 3B,C) while that of other organs showed no significant difference (Figure S3A,B, Supporting Information).

Subsequently, for an accurate assessment of the binding capability of HI@PSeP‐IMTP, photoacoustic computed tomography (PACT) (CalPACT/Union Photoacoustic Technologies, China) was employed to monitor whether these nanoparticles specifically accumulated in the ischemic region of the left ventricular anterior wall (LVAW). To grasp the anatomy of the cardiac cross‐section, we utilized PACT to capture the cross‐section of the heart in a normal mouse (Figure 3D). Then, the targeted effect of HI@PSeP‐IMTP was monitored by PACT. As expected, HI@PSeP‐IMTP showed more excellent targeted effect to the ischemic region of LVAW (Figure 3E,F), which increased by ≈40% compared to the HI@PSeP group on the eighth hour. To further validate that our nanoprobes specifically targeted injured myocardium through IMTP's interaction with cTnI, DiD‐labeled HI@PSeP, and HI@PSeP‐IMTP were employed, and we quantified the targeted efficiency of our nanoprobes by immunofluorescence assay. As shown in Figure S4 (Supporting Information), HI@PSeP‐IMTP exhibited more excellent binding capability toward injured myocardium by interacting with cTnI, which rose by ≈52% compared to HI@PSeP group. To specifically quantify the distribution of nanoprobes in LVAW, ICP/MS was applied and the results showed that selenium increased by ≈57% in LVAW in HI@PSeP‐IMTP group compared to the HI@PSeP group, which further implied that HI@PSeP‐IMTP possessed more excellent targeted capability toward LVAW than HI@PSeP (Figure S5, Supporting Information). Ultimately, we employed transthoracic echocardiography (TTE) to detect the therapeutic efficacy of HI@PSeP and HI@PSeP‐IMTP. As illustrated in Figure S6 (Supporting Information), HI@PSeP‐IMTP exhibited better efficacy in improving cardiac function, including left ventricular ejection fraction (LVEF) and left ventricular fractional shortening (LVFS), left ventricular end‐diastolic diameter (LVDd) and left ventricular end‐systolic diameter (LVDs), which could be attributed to the more excellent targeted capability of HI@PSeP‐IMTP.

Overall, IMTP can effectively enhance the binding capability of the drug delivery system to the region of MIRI. This phenomenon can be monitored by NIR fluorescence imaging technology and PACT in real‐time, and the latter may possess the potential application ability within the clinical setting due to its high ultrasonic resolution and great penetration depth.^[^ ^19^ ^]^

### 2.4 HI@PSeP‐IMTP Mitigates Myocardial Inflammation

**Figure 4:**
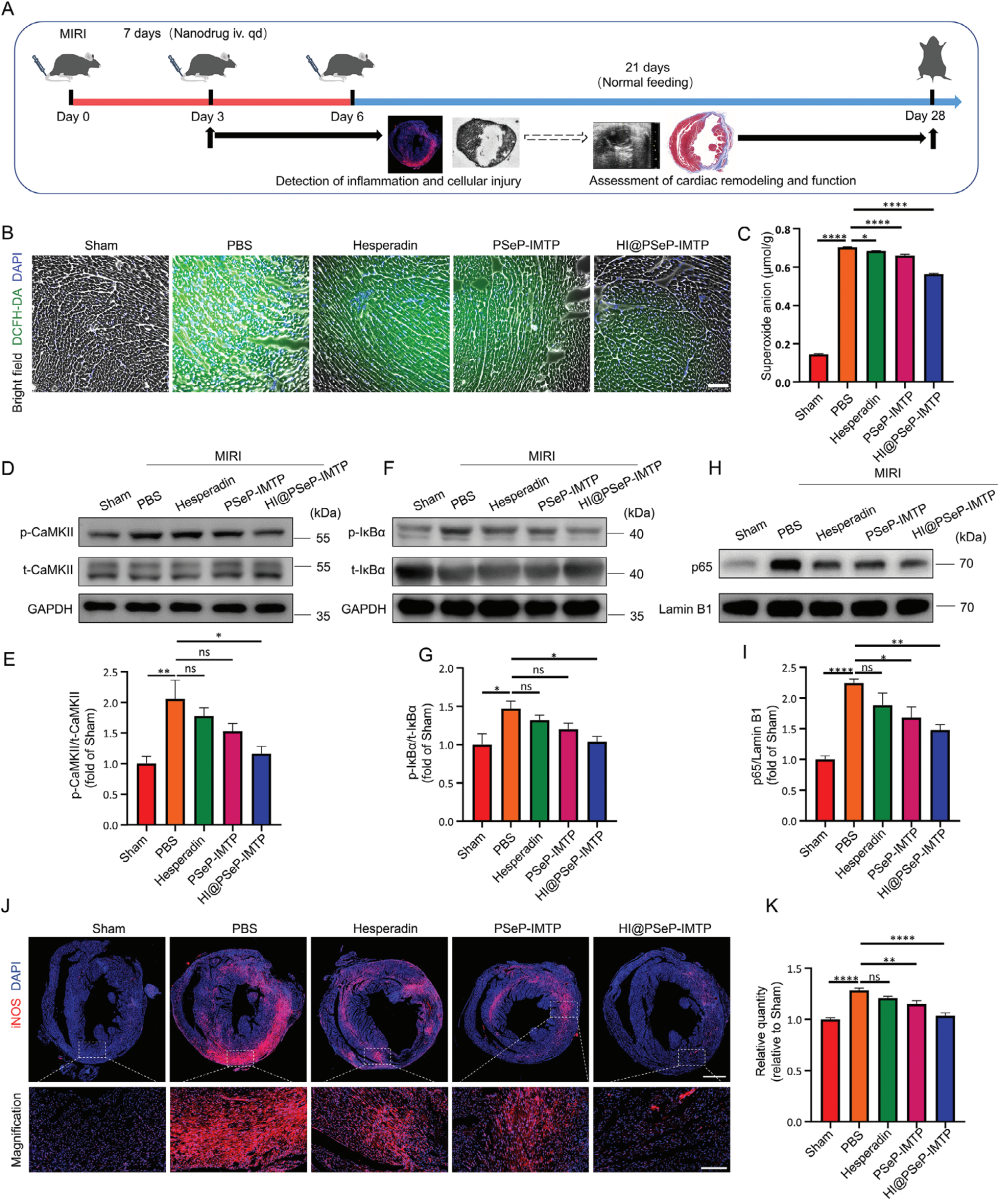
ROS scavenging and suppression of inflammation. A) Schematic illustration of drug administration and the detection of treatment efficacy. The detection of inflammation and cellular injury was on the 3rd day. The assessment of cardiac remodeling and function was on the 28th day. B) Fluorescence imaging was used to observe the expression of ROS in the region of MIRI 3 d after treatments. Scale bar, 100 µm. C) The quantification of superoxide anion in LVAW. D–I) The expression of inflammation‐related proteins was detected by western blot 3 d after treatment. J,K) Fluorescence imaging was applied to discern the distribution of iNOS (M1 macrophages marker, red) in the region of MIRI 3 d after treatment. Scale bar, 1000 µm. Enlarged scale bar, 200 µm. The results were presented as the mean ± SEM (*n* = 7 for the quantification of the expression of superoxide anion and iNOS in LVAW; *n* = 5 for the quantification of the expression of inflammation‐related proteins in LVAW; ns, no significance, ^*^ *p* < 0.05, ^**^ *p* < 0.01, ^***^ *p* < 0.001, ^****^ *p* < 0.0001).

**Figure** **4** A depicted the diagram of the therapeutic protocol and the assessment of treatment efficacy. Considering that ROS and inflammation were major pathogenic factors in MIRI, we principally investigated how the drug delivery system eliminated ROS and ameliorated cardiac inflammation.

To demonstrate the ROS scavenging capacity of HI@PSeP‐IMTP in vivo, a ROS detection assay was employed. As illustrated in Figure 4B, extensive ROS was overproduced by injured cardiac cells in the PBS‐treated MIRI group compared to the Sham group, and the amount of ROS was significantly reduced in the treatment groups, with HI@PSeP‐IMTP group exhibiting the better ROS scavenging ability, which was consistent with the quantification of superoxide anion in ischemic myocardium (Figure 4C). To further investigate the mechanism of HI@PSeP‐IMTP protecting myocardiocytes from MIRI, the expression of inflammation‐associated proteins p‐CaMKII, p‐IκBα, and p65 was detected by western blot. It revealed that the MIRI mice treated with HI@PSeP‐IMTP exhibited the significantly decreased expression of p‐CaMKII (Figure 4D,E), p‐IκBα (Figure 4F,G) and p65 (Figure 4H,I) in the ischemic area compared to the other MIRI group. Specifically, the expression levels of p‐CaMKII, p‐IκBα, and p65 in MIRI region in the HI@PSeP‐IMTP group demonstrated a reduction of 30%, 46%, and 42% respectively compared to that of the PBS group. Subsequently, immunofluorescence staining was performed to identify the distribution of M1 macrophages in the region of MIRI. The result indicated that plentiful M1 macrophages infiltrated into the ischemic cardiac territory, and HI@PSeP‐IMTP possessed the better capacity to attenuate the inflammatory infiltration rather than hesperadin and PSeP‐IMTP (Figure 4J,K).

To sum up, our study demonstrated that HI@PSeP‐IMTP had potent efficacy in ameliorating inflammatory infiltration in the MIRI region through countering the excessive activation of the inflammatory signaling pathway.

### 2.5 HI@PSeP‐IMTP Alleviates Myocardial Damage

It was reported that MIRI induced the overexpression of ROS in the mitochondria of injured cardiac cells, subsequently activating the inflammatory signaling pathway.^[^ ^1^, ^20^ ^]^ Excessive production of ROS and inflammatory proteins combined to damage myocardial cells. Therefore, simultaneous suppression of the overproduction of ROS and inflammatory proteins might attenuate myocardial injury, including mitochondrial injury and DNA damage.

**Figure 5:**
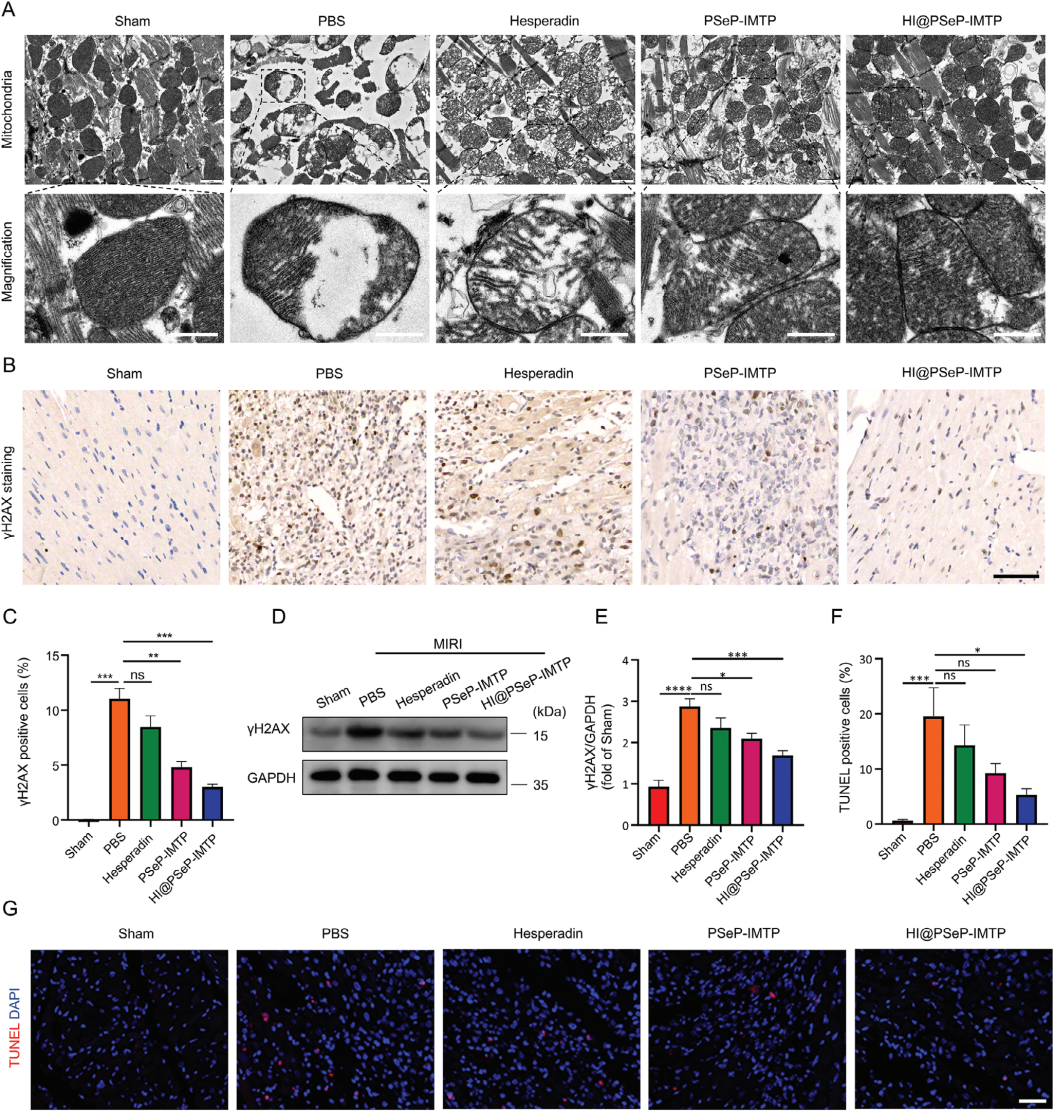
HI@PSeP‐IMTP alleviates cardiac injury 3 d after treatment. A) TEM was used to detect the mitochondrial structure in the MIRI region. Scale bar, 1 µm. Enlarged scale bar, 500 nm. B,C) Immunohistochemical staining of γH2AX in the MIRI region. Scale bar, 60 µm. D,E) The expression of γH2AX in the MIRI region was discerned by western blot. F,G) Myocardial cell death was observed by TUNEL staining. Scale bar, 40 µm. The results were presented as the mean ± SEM (*n* = 7 for the quantification of γH2AX detected by immunohistochemistry assay and the detection of TUNEL in LVAW; *n* = 5 for the quantification of γH2AX detected by Western Blot; ns, no significance, ^*^ *p* < 0.05, ^**^ *p* < 0.01, ^***^ *p* < 0.001, ^****^ *p* < 0.0001).

To gain further insight into the degree of mitochondrial damage, TEM was applied to evaluate the morphology changes of mitochondria in damaged cardiac cells. The results showed that hesperadin, PSeP‐IMTP, and HI@PSeP‐IMTP could effectively mitigate the swelling of mitochondria as well as cristae disruption and loss, especially HI@PSeP‐IMTP performed best (**Figure** **5**A). Notably, the reduction of mitochondrial damage coincided with the alleviation of cardiac sarcomere injury.

Additionally, the expression level of γH2AX, a biomarker of DNA damage, in the MIRI tissue was detected by immunohistochemical staining (Figure 5B,C) and western blot (Figure 5D,E). As expected, HI@PSeP‐IMTP down‐regulated the expression level of γH2AX to the greatest extent, which indicated that the degree of DNA injury in ischemic myocardial cells was the lightest. Ultimately, terminal deoxynucleotidyl transferase‐mediated nick end labeling (TUNEL) assay further confirmed that HI@PSeP‐IMTP protected the most myocardial cells from apoptosis, compared with other MIRI groups (Figure 5F,G).

Overall, these results indicated that HI@PSeP‐IMTP effectively alleviated MIRI‐induced myocardial lesions by attenuating mitochondrial injury, down‐regulating γH2AX expression, and subsequently decreasing the apoptosis rate of cardiac cells.

### 2.6 HI@PSeP‐IMTP Facilitates Cardiac Repair and Ameliorates Myocardial Remodeling

**Figure 6:**
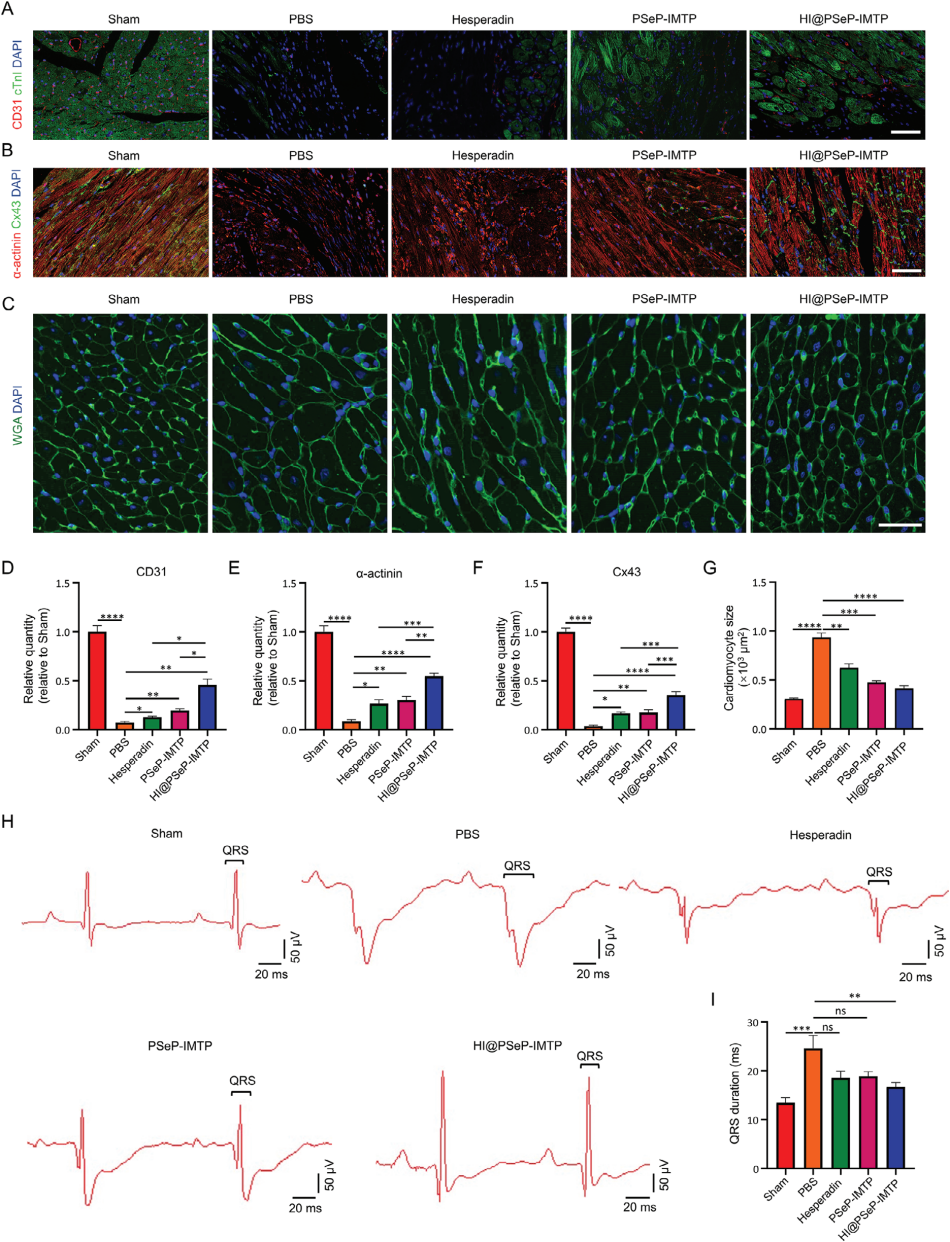
HI@PSeP‐IMTP promotes cardiac repair and alleviates myocardial remodeling in MIRI mice 28 d after treatment. A,B) Immunofluorescence staining was utilized to demonstrate the expression of CD31, Cx43, and α‐actinin in the region of MIRI. Scale bar, 60 µm. C) Myocardial hypertrophy was detected by WGA staining. Scale bar, 40 µm. D–G) CD31, Cx43, and α‐actinin expression levels as well as cardiomyocyte size were quantitatively analyzed. H) ECG was employed to assess the QRS duration. I) Quantitative analysis of QRS duration. The results were shown as the mean ± SEM (*n* = 7; ns, no significance, ^*^ *p* < 0.05, ^**^ *p* < 0.01, ^***^ *p* < 0.001, ^****^ *p* < 0.0001).

To assess whether the regulation of inflammatory response in myocardium undergoing MIRI was conducive to neovascularization, the expression level of CD31 was detected by immunofluorescence assay 3 d after MIRI. Significantly, the results indicated that HI@PSeP‐IMTP facilitated more angiogenesis in the infarct region, compared with other MIRI groups (**Figure** **6**A,D).

As reported, inflammatory stress could disrupt cellular gap junction channels, leading to the loss of gap junction protein connexin 43 (Cx43).^[^ ^21^ ^]^ Immunofluorescence assays revealed that Cx43 proteins of HI@PSeP‐IMTP group in MIRI region were preserved to the greatest extent. Interestingly, the preservation of Cx43 proteins by HI@PSeP‐IMTP was consistent with the protection of α‐actinin proteins, a cardiac cytoskeletal protein involved in intercellular communication^[^ ^22^ ^]^ (Figure 6B,E,F). These results suggested that HI@PSeP‐IMTP contributed to improve cardiac electrical conduction. In addition, wheat germ agglutinin (WGA) staining was used to assess pathological myocardial hypertrophy after MIRI. The findings indicated that HI@PSeP‐IMTP significantly alleviated cardiac hypertrophy, while hesperadin and PSeP‐IMTP had little effect (Figure 6C,G).

To further elucidate the efficacy of HI@PSeP‐IMTP in mitigating myocardial remodeling in vivo, electrocardiogram (ECG) was utilized to analyze the length of the QRS complex, indicative of the extent of myocardial scarring.^[^ ^23^ ^]^ As anticipated, the QRS duration in the HI@PSeP‐IMTP group was shorter than that in the hesperadin and PSeP‐IMTP groups, aligning with the previous findings (Figure 6H,I).

In sum, HI@PSeP‐IMTP demonstrates excellent efficacy in prompting angiogenesis, reducing damage to Cx43 and α‐actinin proteins in the MIRI region as well as countering pathological myocardial hypertrophy, as evidenced by the corresponding QRS duration observed in ECG monitoring.

### 2.7 HI@PSeP‐IMTP Improves Cardiac Function and Alleviates Fibrosis

To further pinpoint the essential role of HI@PSeP‐IMTP in ameliorating the cardiac function of the mice undergoing MIRI, TTE was performed to evaluate the cardiac function 28 d after the administration of PBS, hesperadin, PSeP‐IMTP, and HI@PSeP‐IMTP, respectively. In addition, to clarify there were no significant differences of cardiac function among the groups, the initial ECG and myocardial function after MIRI were assessed as baselines. As illustrated in Figures S7, S8A–E (Supporting Information), there were no significant differences among the PBS group, hesperadin group, PSeP‐IMTP group, and HI@PSeP‐IMTP group on the extent of ST‐segment elevation and indexes of cardiac function.

**Figure 7:**
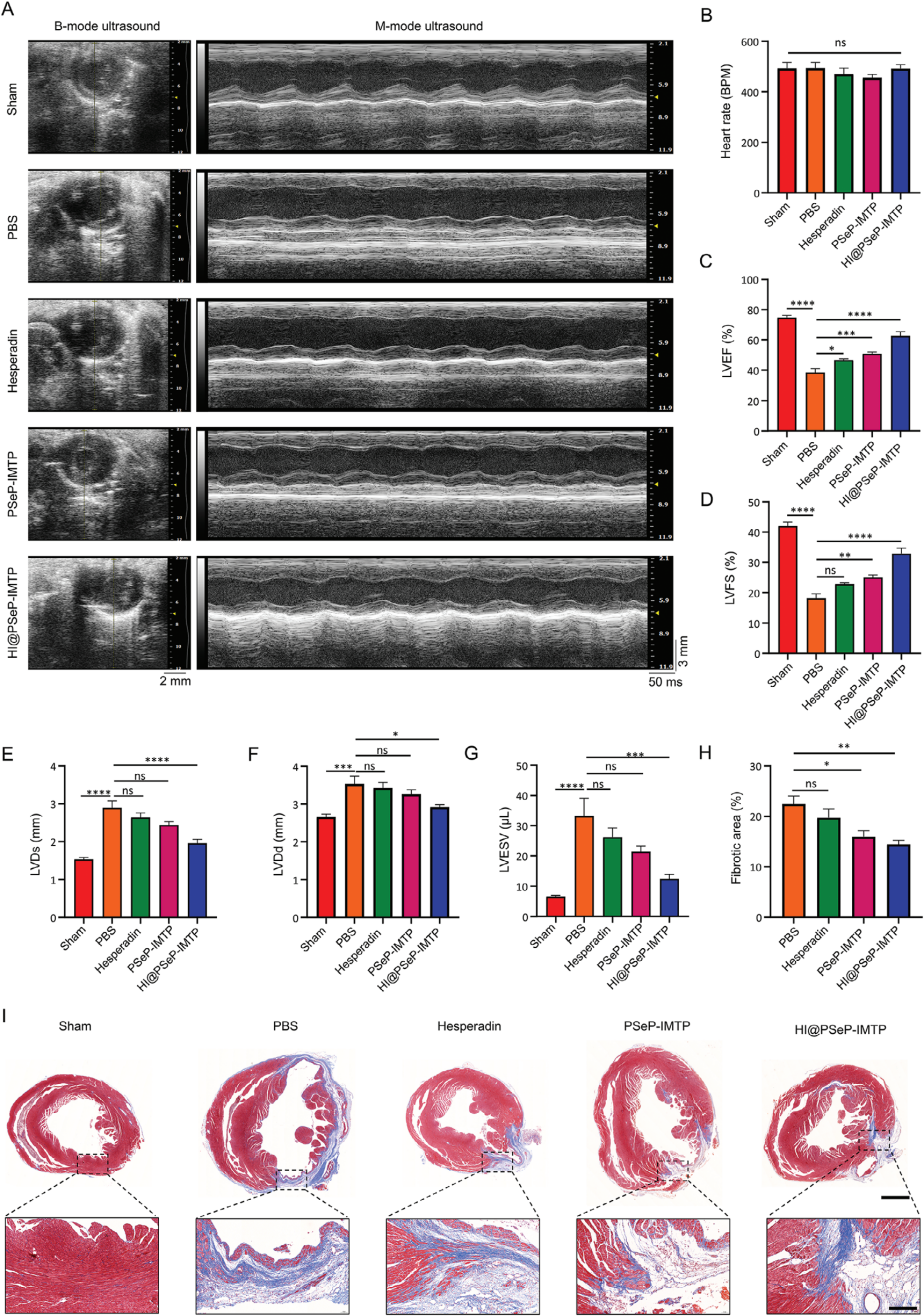
HI@PSeP‐IMTP improves cardiac function and alleviates fibrosis 28 d after treatment. A) TTE images of all groups. Quantitative analysis of Heart rate B), LVEF C), LVFS D), LVDs E), LVDd F) and LVESV G). I) Masson staining was applied to detect the fibrotic area at the level of left ventricular papillary muscles. Scale bar, 1000 µm. Enlarged scale bar, 200 µm. H) Quantitative analysis of the fibrotic area. The results were shown as the mean ± SEM (*n* = 7; ns, no significance, ^*^ *p* < 0.05, ^**^ *p* < 0.01, ^***^ *p* < 0.001, ^****^ *p* < 0.0001).

Subsequently, we conducted TTE assay for each group under the condition of similar heart rate (**Figure** **7**A,B). Significantly, treatment with either hesperadin or PSeP‐IMTP contributed to some improvement of cardiac function, including an increase in LVEF and LVFS as well as a decrease in LVDd, LVDs, and left ventricular end‐systolic volume (LVESV). The therapeutic effect was further enhanced in the group treated with HI@PSeP‐IMTP by combining hesperadin and diselenide bonds, as demonstrated by the better improvement in all indexes of myocardial function (Figure 7C–G). The results indicated that hesperadin and diselenide bonds, a winning combination, synergistically fostered myocardial repair after MIRI. More importantly, these aforesaid results were further supported by the observation of a reduced fibrotic area at the level of left ventricular papillary muscles in mice treated with HI@PSeP‐IMTP (Figure 7H,I).

Taken together, a synergistic integration of hesperadin and diselenide bonds confers additive benefits in terms of infarct size reduction and cardiac function improvement in mice subjected to MIRI.

### 2.8 Biosafety of HI@PSeP‐IMTP

The potential toxicity of HI@PSeP‐IMTP was systematically assessed in C57BL/6J mice. First, we evaluated the blood and biochemical parameters of the mice after treatment, including peripheral blood cell counts as well as liver and kidney function tests. The results indicated no significant differences among the Sham group, PBS group, and HI@PSeP‐IMTP group (Figure S9A–G, Supporting Information). Subsequently, we collected red blood cells from these mice and incubated them with various concentrations of HI@PSeP‐IMTP to assess hemolytic activity. As expected, HI@PSeP‐IMTP did not induce hemolysis of red blood cells (Figure S9H,I, Supporting Information). Ultimately, hematoxylin and eosin (HE) staining was performed to examine the tissue morphology of the liver, spleen, lung, and kidney in different groups after treatment. The results indicated that the tissue morphology in the HI@PSeP‐IMTP group was comparable to that of the Sham and PBS groups, further highlighting the low systemic toxicity of HI@PSeP‐IMTP (Figure S9J, Supporting Information). In a word, HI@PSeP‐IMTP demonstrates excellent biocompatibility and little tissue toxicity.

## 3 Conclusion

In this study, we designed and synthesized a versatile nanocomposite, HI@PSeP‐IMTP, with both diagnostic and therapeutic capabilities. The nanocomposite was functionalized with IMTP to precisely navigate toward the MIRI region and subsequently release cargoes comprising ICG and hesperadin in response to ROS. By taking advantage of ICG, NIR fluorescence imaging and PACT were employed to monitor the accumulation of the nanocomposite in the area of MIRI in real‐time. Moreover, the integration of hesperadin with diselenide bonds orchestrated a synergistic therapeutic effect, concurrently fostering ROS scavenging, mitigating excessive inflammation in the MIRI region, and ultimately ameliorating cardiac function. These findings provide new insights into the development of drugs integrating diagnostic and therapeutic functions tailored for MIRI.

## 4 Experimental Section

### Materials

Dichloromethane (D116146) was purchased from Aladdin Biochemical Technology (Shanghai). Polyvinyl alcohol (PVA) (#9002‐89‐5) was purchased from Shanghai yuanye Bio‐Technology Co., Ltd. Hesperadin (#422513‐13‐1) and Hoechst 33342 (#875756‐97‐1) were purchased from MedChemExpress. CSTSMLKAC (IMTP) (AP231031) was obtained from Apeptide (Shanghai, China). PLGA‐Se‐Se‐PEG‐NHS (R‐CW‐PS012) with an average molecular weight of 10 000 Da and lipophilic ICG (R‐TE‐157) were obtained from Xi'an ruixi Biological Technology Co., Ltd. Annexin V‐FITC/PI Apoptosis Detection Kit (AD10) and Cell Counting Kit‐8 (CCK‐8) (CK04) were obtained from Dojindo Laboratories. ROS Assay Kit (G1706), Calcein‐AM/PI double staining kit (G1707), TMR (Red) Tunel Cell Apoptosis Detection Kit (G1502), iF488‐Wheat Germ Agglutinin (G1730), iNOS antibody (GB11119‐100), cTnI antibody (GB122408‐100), α‐actinin antibody (GB111556‐100), Cx43 antibody (GB12234‐100), DAPI (G1012) and Masson staining kit (G1006) were obtained from Servicebio. H_2_O_2_ (#7722‐84‐1) was obtained from Sigma–Aldrich. DiD (#127274‐91‐3) was purchased from Xi'an QiYue Biology. HE staining kit (E‐IR‐R117) was obtained from Elabscience Biotechnology Co., Ltd. CD31 antibody (#M1511‐8), Lamin B1 antibody (#ET1606‐27), p65 antibody (#ET1603‐12), p‐CaMKII antibody (#HA721794), CaMKII antibody (#ET1704‐37), p‐IκBα antibody (#ET1609‐78), IκBα antibody (#ET1603‐6), γH2AX antibody (#ET1602‐2) and GAPDH antibody (#HA721136) were obtained from HUABIO.

### Cell Lines and Animals

Human ventricular cardiomyocyte cell lines, AC16, were obtained from Cellcook (Guangzhou, China). The AC16 cardiomyocytes, grown in Dulbecco's modified Eagle's medium (DMEM)/ F12 containing 10% FBS (Gibco), 50 units of penicillin, and 50 µg of streptomycin per mL, were incubated in a humidified incubator at 37 °C with 5% CO_2_. Male C57BL/6J mice (6–8 weeks) and male Balb/c mice (6–8 weeks) were obtained from Guangdong Medical Laboratory Animal Center. It is worth mentioning that male Balb/c mice were only employed to conduct PACT for avoiding melanin influence while C57BL/6J mice were applied in all other animal experiments. In this study, all animal experiments were authorized by the Animal Care and Use Committee of Guangdong Provincial People's Hospital (approval number: KY‐N‐2022‐122‐01).

### Preparation and Characterization of HI@PSeP‐IMTP

The core of HI@PSeP‐IMTP was synthesized by ultrasonic emulsification‐solvent evaporation method.^[^ ^24^ ^]^ Briefly, 20 mg PLGA‐Se‐Se‐PEG‐NHS was dissolved in 3 mL dichloromethane and 1 mL ultrapure water was added into this mixture. Whereafter, the mixture was sonicated by an ultrasonic cell disruption system (BILON, Shanghai, China) at the temperature of 4 °C and a power of 455 W for 10 min. Furthermore, 7 mL 5% PVA was added into the initial product and sonicated in the same conditions. To get rid of dichloromethane, vacuum rotatory evaporator was employed at 25 °C for 5 min. To realize PLGA‐Se‐Se‐PEG‐NHS coupled with IMTP, they were jointly added into PBS (pH 8.0) at the ratio of 1.5:1 and stirred for 6 h at room‐temperature. The product, PSeP‐IMTP, was recovered by centrifugation (5000 rpm, 10 min) and subsequently washed three times by ultrapure water. To incorporate cargoes into the nanoparticles, 1 mg ICG and 500 µg hesperadin were added into the PBS containing 10 mg PSeP‐IMTP nanoparticles. The mixture underwent ultrasonic incubation at 40 kHz for 30 min and was subsequently stirred for 6 h at room‐temperature. The final product, HI@PSeP‐IMTP, was washed three times by ultrapure water and recovered by centrifugation (5000 rpm, 10 min).

Fourier transform infrared Spectrometer (Bruker INVENIO‐R, Germany) was used to assess whether HI@PSeP‐IMTP possessed the characteristic peak of IMTP. The morphology of HI@PSeP and HI@PSeP‐IMTP was monitored by TEM. Litesizer 500 (Anton Paar, Austria) was used to analyze the diameter, surface potential, and PDI of nanoparticles. The elemental distribution of HI@PSeP‐IMTP was measured by energy dispersive spectrometer mapping (JEOL JEM‐F200, Japan). To verify whether HI@PSeP‐IMTP was equipped with an aggregation‐caused‐quenching effect, Multimode Reader (BioTek) was utilized to measure the fluorescence intensity of free ICG and HI@PSeP‐IMTP in the emission wavelength range of 700–850 nm. To discern the ROS‐responsive property of HI@PSeP‐IMTP, the nanocomposite was dissolved in PBS with various concentrations of H_2_O_2_ and then the controlled release of cargoes was measured by IVIS spectrum in vivo imaging system (PerkinElmer, USA) and Multimode Reader in the emission wavelength range of 700–850 nm. To detect the cumulative release of cargoes from HI@PSeP‐IMTP, 100 µg HI@PSeP‐IMTP was respectively added into 200 µL PBS and 200 µL 1 mM H_2_O_2_ at 37 °C for 1 h. Subsequently, the nanoparticles were incubated with 10% FBS. Multimode Reader was employed to detect the release of cargoes at different time points. To exhibit the ROS scavenging capacity of HI@PSeP‐IMTP, the different concentrations of the nanocomposite were dissolved in PBS with 500 µmol L^−1^ H_2_O_2_ at 37 °C for 1 h and then DCFH‐DA was used to detect the residual ROS of each group by IVIS spectrum in vivo imaging system and multimode reader in the emission wavelength range of 500–600 nm.

### Establishment of OGD/R Model In Vitro

AC16 cardiomyocytes were cultured in a humidified incubator under normal conditions for 48 h. Then, the cells were incubated with glucose‐free DMEM (Procell, PM150273) in an anaerobic incubator at 37 °C with 1% O_2_ and 5% CO_2_ for 8 h and subsequently grown under normal conditions for 16 h.

### Targeted and Therapeutic Capability of HI@PSeP‐IMTP In Vitro

To evaluate the binding capability of HI@PSeP‐IMTP to OGD/R‐treated AC16 cardiomyocytes, the cells (2 × 10^4^ per well) were inoculated in 96‐well plates. After OGD, the cells were grown with high‐glucose DMEM containing HI@PSeP (2 µg mL^−1^) or HI@PSeP‐IMTP (2 µg mL^−1^) respectively. Herein, it is worth noting that the ICG of HI@PSeP and HI@PSeP‐IMTP was replaced by DiD for fluorescence microscopy imaging. Ultimately, the nucleuses of AC16 were stained with Hoechst 33342 and THUNDER Imaging System (Leica, Germany) was used to assess the binding capability of HI@PSeP‐IMTP through fluorescence intensity of DiD in the cells 12 h after reoxygenation.

To discern the cardiomyocyte toxicity of HI@PSeP‐IMTP, AC16 cells were incubated with different concentrations of the nanocomposite for 24 h. To evaluate the therapeutic impact of HI@PSeP‐IMTP on AC16 cardiomyocytes suffering from OGD/R, AC16 cells were cultured with different concentrations of HI@PSeP‐IMTP at the onset of reoxygenation for 16 h. The cardiomyocyte toxicity and therapeutic efficacy of HI@PSeP‐IMTP were assessed by CCK‐8 assay. Specifically, the initial culture medium was replaced by a CCK‐8 working solution and the cells were incubated in normal conditions for 2 h. Spark Multimode Reader (TECAN, Switzerland) was employed to discern the absorbance of each well at 450 nm.

The synergistic efficacy of hesperadin and diselenide bonds was validated by incubating OGD/R‐treated AC16 cells with hesperadin (0.07 µg mL^−1^), PSeP‐IMTP (2 µg mL^−1^) or HI@PSeP‐IMTP (2 µg mL^−1^) respectively, at the onset of reoxygenation for 16 h. Subsequently, therapeutic effectiveness was elucidated through the conduct of CCK‐8 assay, apoptosis assay, ROS detection assay, and Calcein‐AM/PI double staining assay. To conduct apoptosis assay, AC16 cells in 96‐well plates of each group after treatment were resuspended in 100 µL binding buffer with 1 µL of Annexin V‐FITC and PI per well for 10 min at room‐temperature. Subsequently, the apoptotic rate of AC16 cells was detected by a CytoFLEX S (Beckman Coulter, USA). To discern the ROS expression levels of each group, AC16 cells in 96‐well plates of each group after treatment were incubated with DCHF‐DA dye (10 µmol L^−1^) for 20 min in a humidified incubator under normal conditions. Afterward, the cells were washed by PBS and THUNDER Imaging System was applied to observe the ROS expression levels of each group. AC16 cells in 96‐well plates of each group after treatment were incubated with working solution of Calcein AM and PI (100 µL per well) for 15 min and washed by PBS. Subsequently, the label cells were observed by THUNDER Imaging System.

### MIRI Model

MIRI surgery was conducted as previously described.^[^ ^25^ ^]^ Specifically, mice (6–8 weeks old) were anesthetized with 1.5% isoflurane inhalation using a rodent ventilator (Penlon Sigma Delta, UK). Animals were placed on a heating pad in a supine position to maintain the body temperature at 37 °C. The skin in the left side of the chest was cut open and the pectoralis muscles were separated by blunt dissection to expose the fourth intercostal space. Subsequently, a small hole was made in the appropriate position and the heart was extruded through the hole. The left anterior descending branch of the coronary artery was found out and then ligated by a 6–0 silk suture 2–3 mm from the origin with a slipknot, after which the heart was immediately returned to the original position, followed by expelling air from the left lung and closing the wound in layers. The slipknot was removed after 1 h. Sham group mice underwent the identical surgical protocol, but without ligating the left anterior descending branch. In the end, whether MIRI model succeeded was evaluated by ST‐segment elevation in ECG.

Once the MIRI model was established successfully, the mice were randomly divided into the Sham, PBS, hesperadin, PSeP‐IMTP, and HI@PSeP‐IMTP group (*n* = 20 per group). Then the mice were intravenously treated with PBS, hesperadin (0.3556 mg kg^−1^), PSeP‐IMTP (10 mg kg^−1^) and HI@PSeP‐IMTP (10 mg kg^−1^) every day from the moment of successful MIRI modeling to the sixth day.

### Binding Capability of HI@PSeP‐IMTP

The IVIS spectrum in vivo imaging system and PACT were employed to pinpoint the targeted ability of HI@PSeP‐IMTP to the MIRI region as previously reported.^[^ ^26^ ^]^ In brief, the mice subjected to MIRI were administered with PSeP‐IMTP (2 mg kg^−1^) or HI@PSeP‐IMTP (2 mg kg^−1^) respectively, after which the region of MIRI was monitored by IVIS spectrum in vivo imaging system and PACT with a selection of 780 nm excitation at different time points.

To further validate the nanoprobes specifically target injured myocardium, DiD‐labeled HI@PSeP (2 mg kg^−1^) and HI@PSeP‐IMTP (2 mg kg^−1^) were employed. The mice were euthanized 8 h after administration of the nanoprobes, and the heart tissues fixed in 4% paraformaldehyde were embedded in paraffin and sliced into 3.5‐µm thick sections at the level of left ventricular papillary muscles. The sections were stained with primary antibodies, cTNI antibody, overnight at 4 °C, followed by staining with corresponding secondary antibody and DAPI. Super Resolution Microscope (N‐SIM) was applied to detect the targeted efficiency of the nanoprobes.

To quantify the biodistribution of nanoparticles specifically, ICP/MS was employed to detect the quantity of selenium in major organs on the eighth hour after administration.

### Immunofluorescence Staining and Immunohistochemistry

On the third day after therapy, C57BL/6J mice were euthanized and perfused with PBS from the left ventricle to eliminate blood in heart tissue. Afterward, the hearts were harvested and fixed with liquid nitrogen (only for ROS detection assay) or 4% paraformaldehyde. To conduct ROS detection assay, the heart tissues were sliced into 7‐µm thick sections at the level of left ventricular papillary muscles by Leica CM1950 (Leica, Germany). The sections were stained with ROS Assay Kit (Servicebio, China) and incubated at 37 °C for 30 min, followed by washing with PBS. Ultimately, the sections were sealed with an antifade mounting medium for fluorescence (BL739A, Biosharp) and subsequently observed by the THUNDER Imaging System.

The heart tissues fixed in 4% paraformaldehyde were embedded in paraffin and sliced into 3.5‐µm thick sections at the level of left ventricular papillary muscles by a microtome (RM2016, Leica, Germany). The sections were stained with primary antibodies, including iNOS antibody, cTnI antibody, CD31 antibody, α‐actinin antibody, and Cx43 antibody, overnight at 4 °C, followed by staining with corresponding secondary antibody and DAPI. The expression of these proteins in the heart tissues was captured by 3DHISTECH (Pannoramic MIDI). Additionally, the sections stained with TMR (Red) Tunel Cell Apoptosis Detection Kit and iF488‐Wheat Germ Agglutinin were processed as mentioned before except incubated with the secondary antibody. Similarly, the sections used for immunohistochemical assay were stained with γH2AX antibody, secondary antibody, and hematoxylin but without DAPI.

### Western Blot Assay

As reported before,^[^ ^1b^ ^]^ the tissues of the MIRI region in LVAW were obtained and immersed in RIPA lysis buffer containing phosphatase and protease inhibitor cocktail. Subsequently, the tissues were lysed by Freezer Mixer (LUKYM‐I) (−20 °C, 70 Hz, 90 s) to gain the proteins, and the tissue fragments were removed by centrifugation (4 °C, 10 000 rpm, 10 min). Then the proteins were separated by 10% or 12% SDS‐PAGE gels and transferred to PVDF membranes, which were blocked with protein‐free rapid blocking solution (G2052‐500ML, Servicebio) and incubated with corresponding antibodies. Ultimately, chemiluminescence detection system (LAS 500) was applied to detect the protein bands.

### TEM

As reported previously,^[^ ^27^ ^]^ the tissues of the MIRI region in LVAW were fixed by 2.5% glutaraldehyde for 2 h. Then the tissues were washed with PBS and immobilized by 1% buffered osmium for 2–3 h, followed by gradient dehydration, embedment, and solidification. Ultimately, the tissues were cut to 65 nm‐thick blocks and stained with 3% uranyl acetate‐lead citrate. A transmission electron microscope (JEM‐1400Flash, Japan) was used to observe the morphology of mitochondria.

### TTE

The cardiac function of all mice was monitored by an ultrasonic imager (Vevo 2100, VisualSonics, Canada) under 1% isoflurane at different time points. M‐mode and B‐mode imaging were obtained in the short‐axis view of the left ventricle at the level of the papillary muscles, which could measure the indexes of cardiac function, including heart rate, LVEF, LVFS, LVDs, LVDd, and LVESV. The data were averaged from 3.5 cardiac cycles and analyzed blindly.

### Masson Staining and HE Staining

C57BL/6J mice were euthanized and the vital organs, including heart, liver, spleen, lung, and kidney, were harvested and fixed with 4% paraformaldehyde. As reported previously,^[^ ^27^ ^]^ Masson Three‐Color Kit (Servicebio, G1006) was employed to assess the fibrosis degree of myocardium 4 weeks after MIRI modeling, and HE Staining Kit (Servicebio, G1005) was applied to evaluate whether HI@PSeP‐IMTP induced the injury of liver, spleen, lung, and kidney 7 d after MIRI.

### Statistical Analysis

The data were presented as the mean ± SEM and the corresponding experiments were performed at least three times. GraphPad Prism 7 software was applied to analyze the data by the Student's *t*‐test or one‐way ANOVA. Differences were exhibited as ^*^ *p* < 0.05, ^**^ *p* < 0.01, ^***^ *p* < 0.001 and ^****^ *p* < 0.0001.

## Conflict of Interest

The authors declare no conflict of interest.

## Author Contributions

X.M. and Z.F. contributed equally to this work. X.M. was responsible for conceptualization, investigation, methodology, and project administration. Z.F. was responsible for conceptualization and data curation. J.P. was responsible for methodology. L.N. was responsible for supervision. All authors also wrote, reviewed, and edited the final manuscript.

## Supporting information



Supporting Information

## Data Availability

The data that support the findings of this study are available from the corresponding author upon reasonable request.
