# Supplementary material for: Ischemic Area‐Targeting and Self‐Monitoring Nanoprobes Ameliorate Myocardial Ischemia/Reperfusion Injury by Scavenging ROS and Counteracting Cardiac Inflammation
Source: Adv Sci (Weinh). 2025 Jan 22;12(11):2414518. doi: 10.1002/advs.202414518 (PMC11923900; doi:10.1002/advs.202414518)
Supplement: Supplementary file 1 — Supporting Information [file ADVS-12-2414518-s001.doc]

**Supporting Information**

**Ischemic Area-targeting and Self-monitoring Nanoprobes Ameliorate Myocardial Ischemia/Reperfusion Injury by Scavenging ROS and Counteracting Cardiac Inflammation**

Xiaobin Ma^1, 2†^, Zhijin Fan^2, 3†^, Jingyan Peng^2^, and Liming Nie^1, 2*^

1. Department of Cardiology, Guangdong Cardiovascular Institute, Guangdong Provincial People’s Hospital, Guangdong Academy of Medical Sciences, Guangzhou, 510080, China.

2. Medical Research Institute, Guangdong Provincial People's Hospital (Guangdong Academy of Medical Sciences), Southern Medical University, Guangzhou, 510080, China.

3. Institute for Engineering Medicine, Kunming Medical University, Kunming, 650500, China.

*Corresponding Author: nieliming@gdph.org.cn.

†These authors contributed equally to this work.


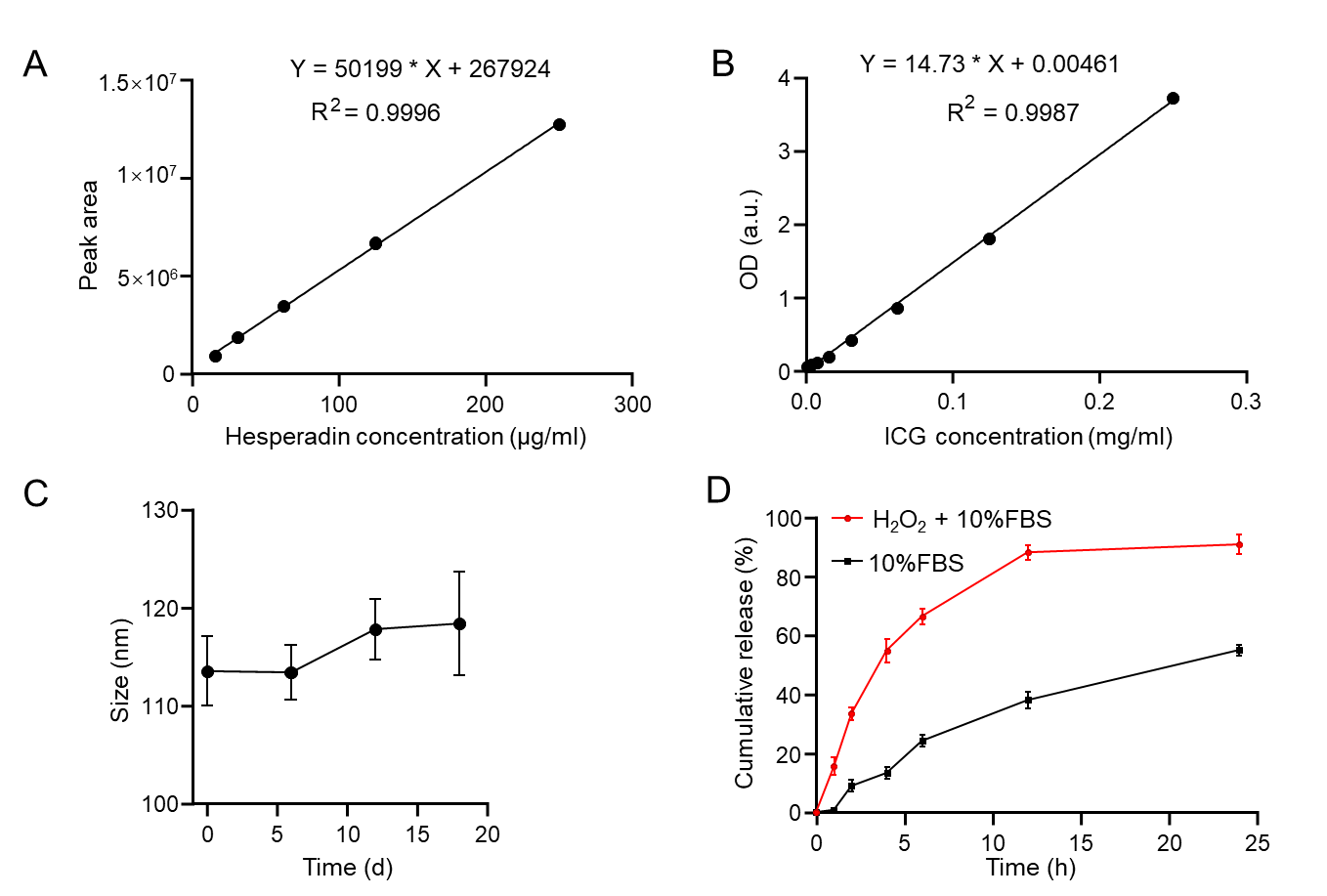


Figure S1. The standard curve of cargoes in the nanoprobes and the performance assessment of nanoprobes. A. Standard curve of hesperadin. B. Standard curve of ICG. C. Stability of HI@PSeP-IMTP in PBS at 4 ℃. D. Cumulative release of cargoes from HI@PSeP-IMTP in 10% FBS with or without H_2_O_2_. The results were shown as the mean ± SEM.


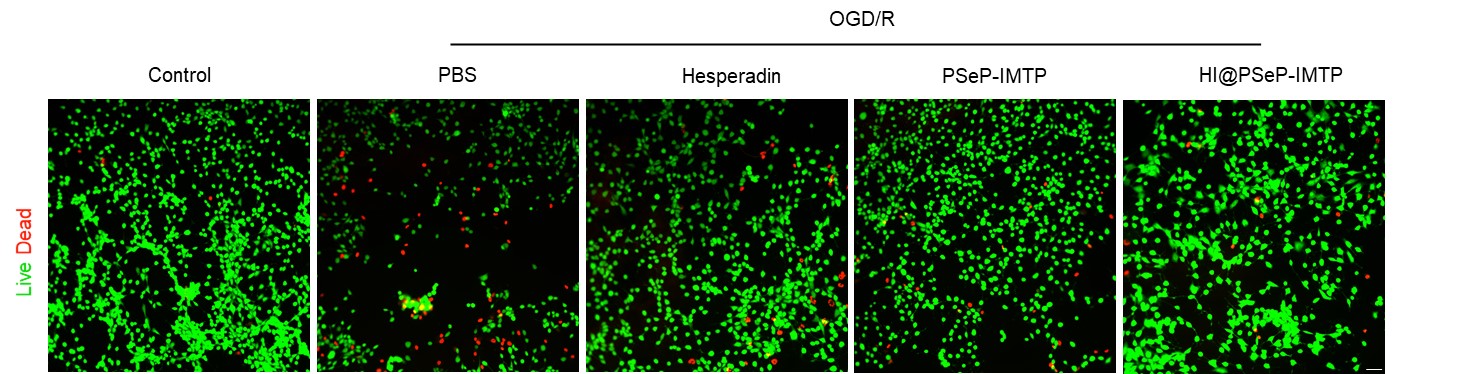


**Figure S2.** **The assessment of the therapeutic capability of HI@PSeP-IMTP in vitro.** LIVE/DEAD cells assay was used to assess the effects of different treatments. Scale bar = 75 μm.


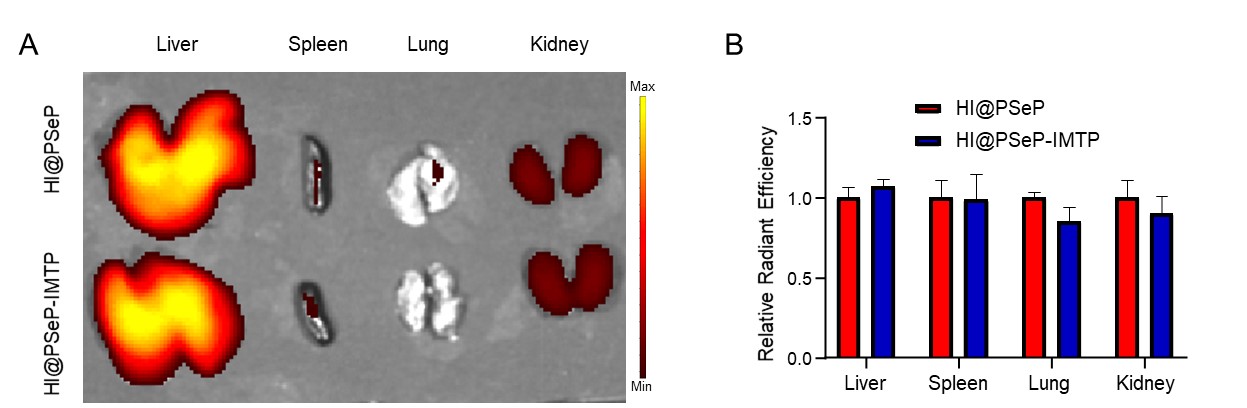


Figure S3. The distribution of nanoprobes in vital organs. A. NIR fluorescence imaging was employed to assess the distribution of nanoprobes in liver, spleen, lung and kidney. B. Relative radiant efficiency of different organs was quantified. The results were shown as the mean ± SEM (n = 3).


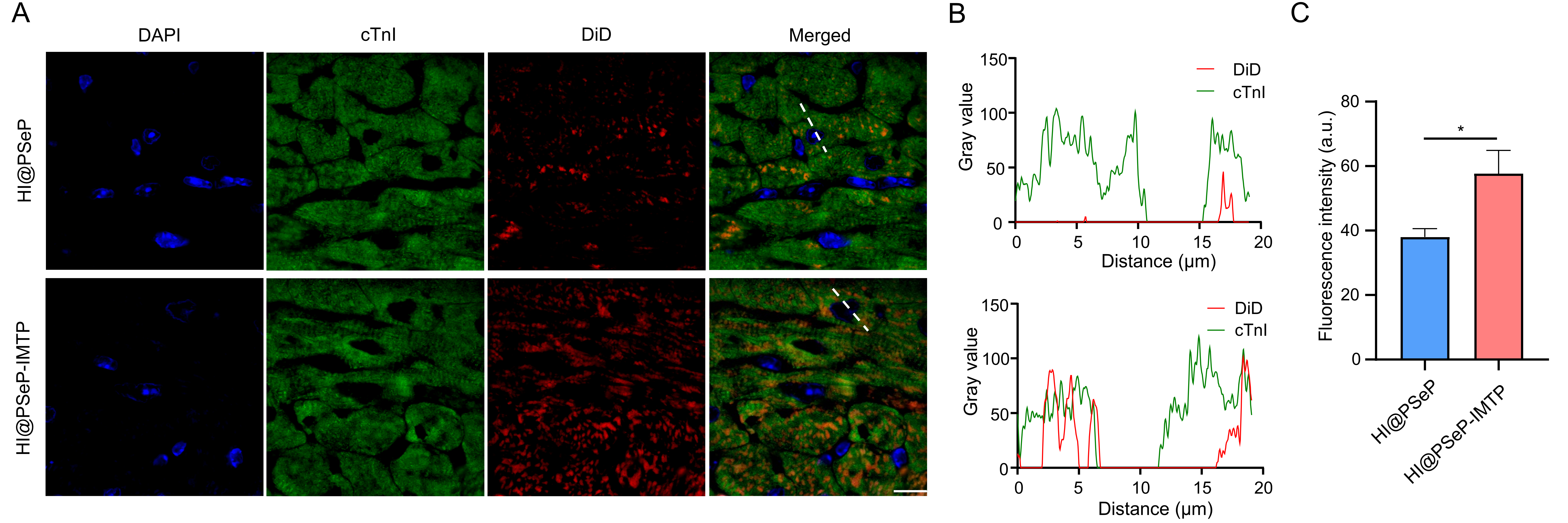


**Figure S4. The detection of nanoprobes in the injured myocardium 8 h after administration. A.B.** Immunofluorescence was applied to assess the colocalization of nanoprobes and exposed cTnI in the injured myocardium. Scale bar = 10 μm. **C.** The aggregation of DiD-labeled HI@PSeP and HI@PSeP-IMTP in injured myocardium was quantified by fluorescence intensity. The results were shown as the mean ± SEM (n = 5; * p ＜ 0.05).

**
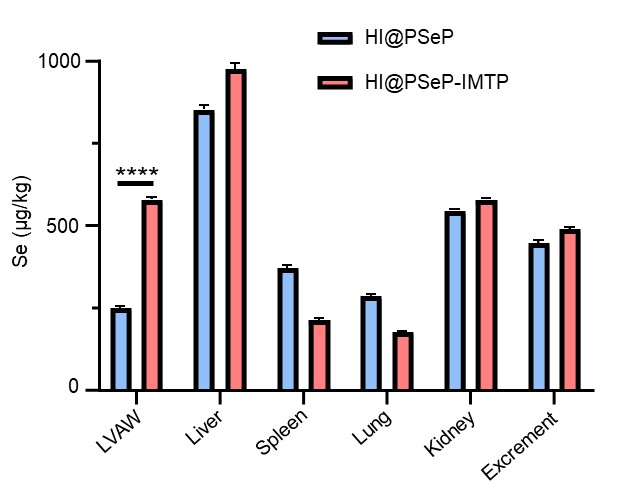
**

**Figure S5. The quantitative biodistribution of nanoprobes in vivo 8 h after administration.** ICP/MS was used to detect the biodistribution of Se in vivo 8 h after administration. The result was shown as the mean ± SEM (n = 3; **** p ＜ 0.0001).


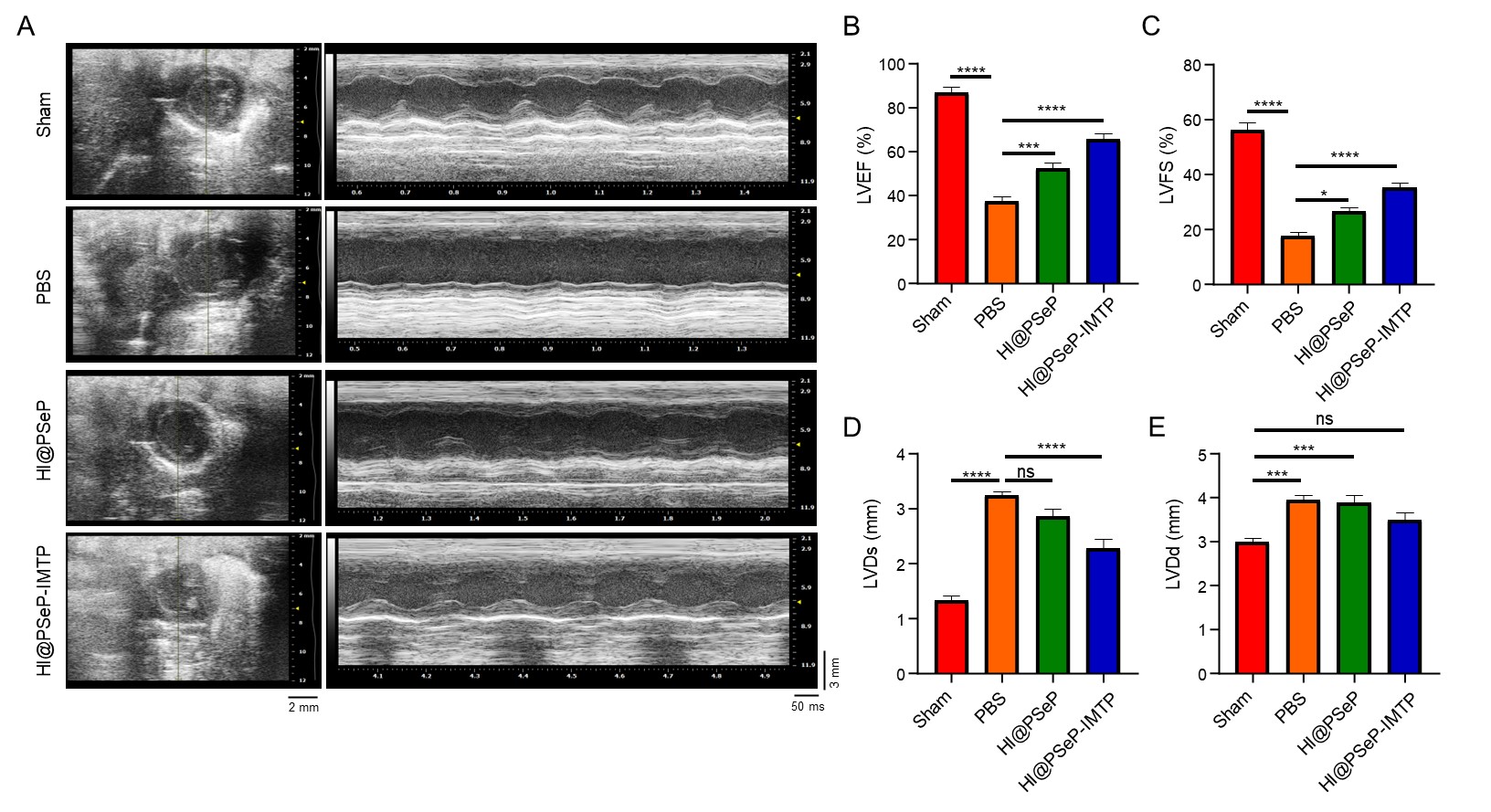
**Figure S6. The assessment of the therapeutic effect of nanoprobes with or without targeted capability 28 d after MIRI. A.** TTE images of all groups. Quantitative analysis of LVEF **(B)**, LVFS **(C)**, LVDs **(D)**, LVDd **(E)**. The results were shown as the mean ± SEM (n = 7; ns, no significance, * p ＜ 0.05, *** p ＜ 0.001, **** p ＜ 0.0001).


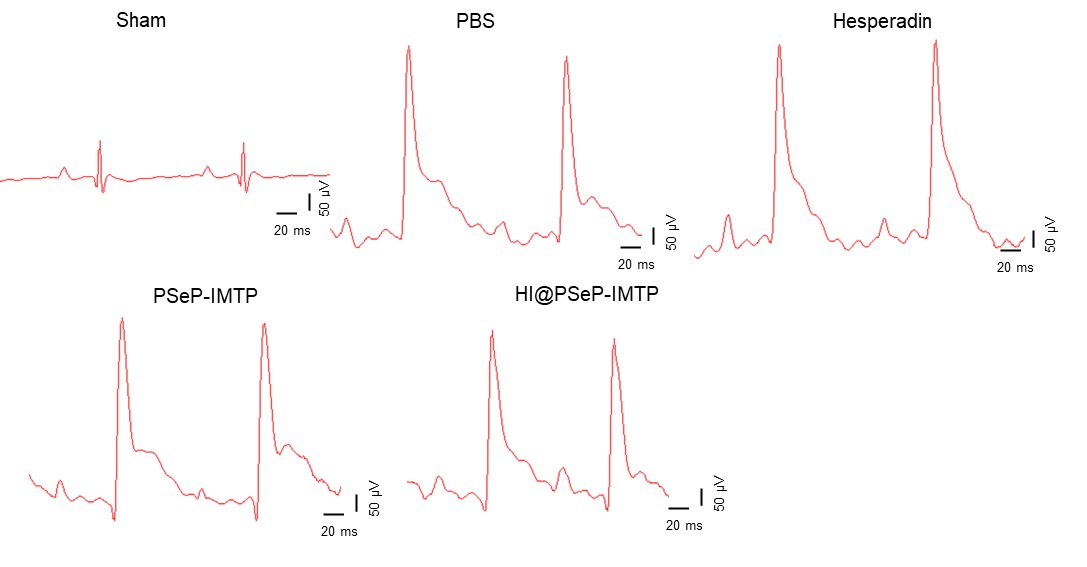


**Figure S7. The ECG was monitored in different groups 5 min after modelling.**


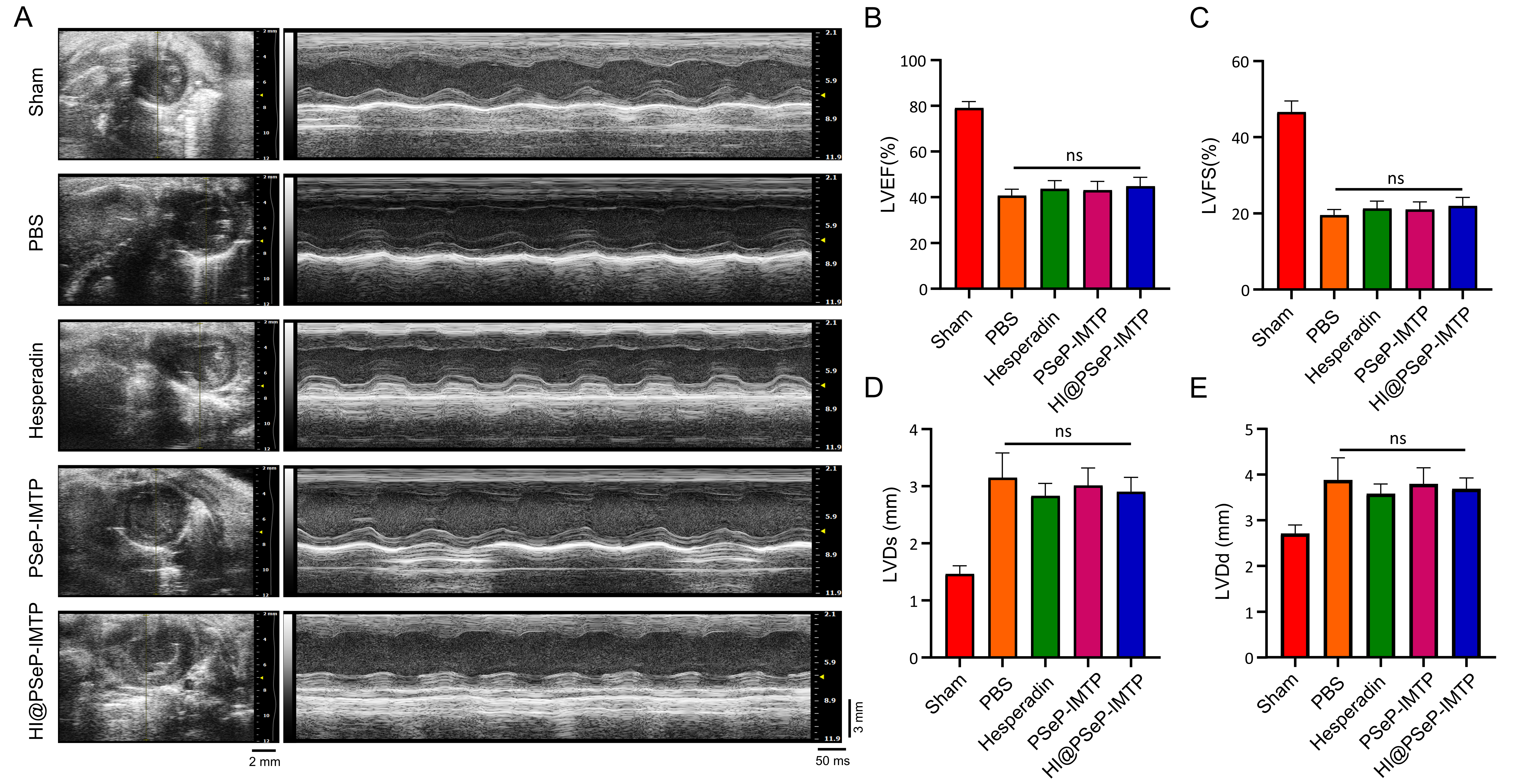


**Figure S8.** **The baseline of cardiac function in different groups was detected 6 h after MIRI. A.** TTE images of all groups. Quantitative analysis of LVEF **(B)**, LVFS **(C)**, LVDs **(D)**, LVDd **(E)**. The results were shown as the mean ± SEM (n = 7; ns, no significance).

**
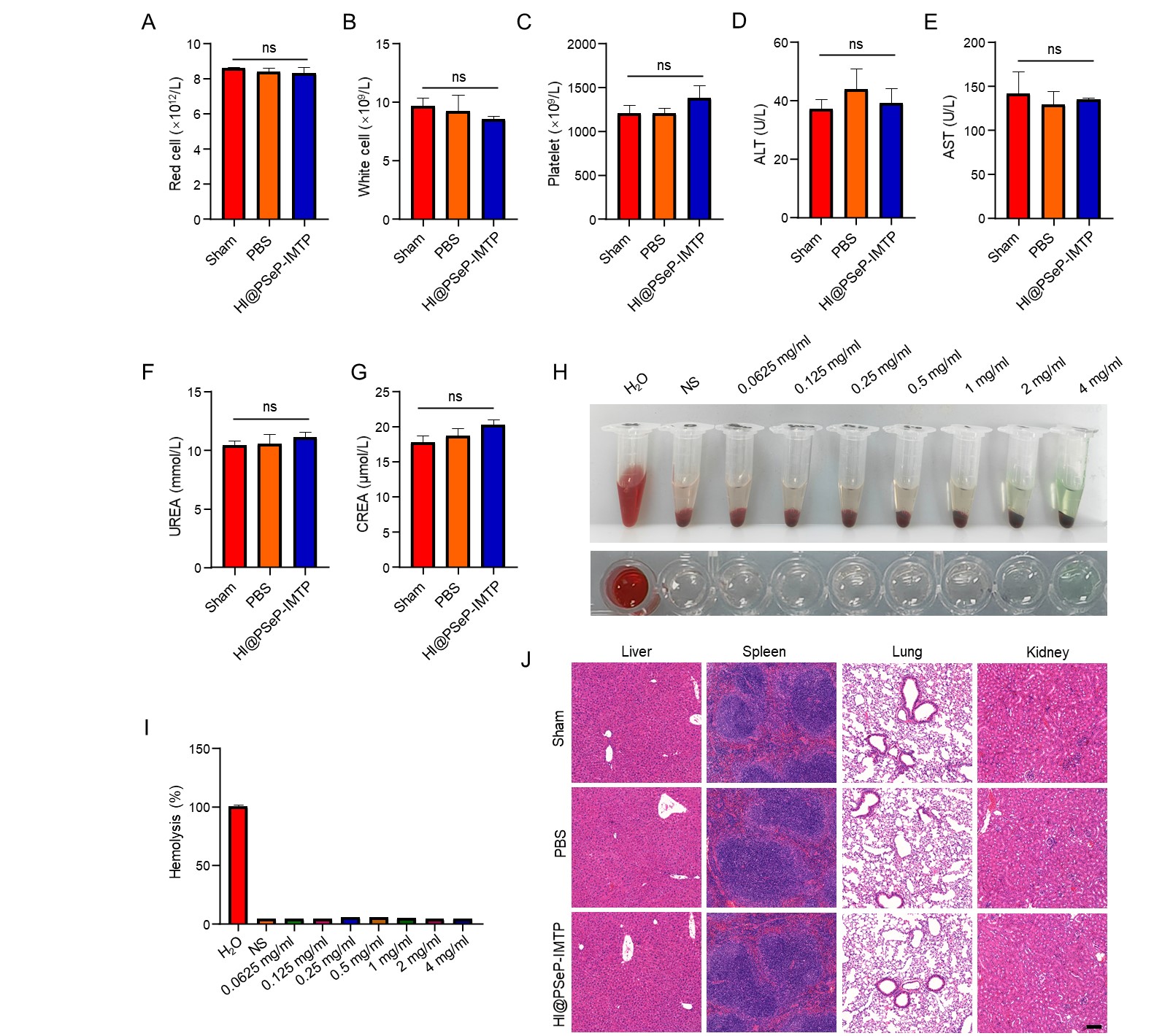
**

**Figure S9. Biosafety of HI@PSeP-IMTP.** Quantitative analysis of the biosafety of HI@PSeP-IMTP by blood routine examination **(A, B, C)**, liver function **(D, E)** and kidney function **(F, G)**. **H.I.** Hemolytic test was applied to detect the hemolysis rate of HI@PSeP-IMTP. **J.** HE staining assay was conducted to detect the effect of HI@PSeP-IMTP on vital organs. Scale bar = 100 μm. The results were shown as the mean ± SEM (n = 3; ns, no significance).
